# Regulatory emotional self-efficacy and psychological distress among medical students: multiple mediating roles of interpersonal adaptation and self-acceptance

**DOI:** 10.1186/s12909-022-03338-2

**Published:** 2022-04-15

**Authors:** Xuemin Zhang, Heng Yue, Junfang Sun, Min Liu, Cuiyun Li, Hugejiletu Bao

**Affiliations:** 1 School of Psychology, Inner Mongolia Normal University, 81 Zhaowuda Road, Saihan District, Hohhot, 010022 China; 2 School of Humanity, Baotou Medical College, No.31 Jianshe Road, East District, Baotou, 014040 China; 3 School of Physical Education, Inner Mongolia Normal University, 81 Zhaowuda Road, Saihan District, Hohhot010022, China

**Keywords:** Interpersonal adaptation, Psychological distress, Regulatory emotional self-efficacy, Self-acceptance, Medical students, DASS-21

## Abstract

**Background:**

Psychological distress (depression, anxiety and stress) is more common among medical students than in the general population, and is an important cause of insomnia, internet addiction, substance abuse, decreased academic performance and increased suicidality in medical students.

**Methods:**

To examine the mechanism by which regulatory emotional self-efficacy affects medical students' psychological distress, a questionnaire of 539 medical students using an interpersonal adaptability scale, regulatory emotional self-efficacy scale, self-acceptance scale and depression-anxiety-stress scale was conducted.

**Results:**

① Regulatory emotional self-efficacy, interpersonal adaptability and self-acceptance are positively correlated, but they are negatively correlated with psychological distress. ② The mediation model shows that interpersonal adaptation and self-acceptance are the mediation variables of the effect of regulatory emotional self-efficacy on psychological distress, and the total mediation effect value is -0.37, accounting for 86.05% of the total effect (-0.43). Specifically, the effect involves three paths: first, regulatory emotional self-efficacy indirectly affects psychological distress through interpersonal adaptation (effect value-0.24); second, regulatory emotional self-efficacy indirectly affects psychological distress through interpersonal adaptation and self-acceptance (effect value-0.08); and third, regulatory emotional self-efficacy indirectly affects psychological distress through self-acceptance (effect value -0.05).

**Conclusions:**

Interpersonal adaptation and self-acceptance have a significant mediating effect between regulatory emotional self-efficacy and psychological distress, and the chain mediating effect of interpersonal adaptation and self-acceptance is also significant.

## Background

In recent years, mental health problems in medical students have become more common. Long-term medical education, strict examination systems, intensive professional learning tasks and tense doctor–patient relationships make medical students more anxious and depressed than others of the same age [1]. Psychological distress involves a state of emotional pain and a mixed state of negative emotions such as depression and anxiety, sometimes accompanied by physical symptoms [2]. Researchers usually use the Depression-Anxiety-Stress Scale (DASS-21) to assess the degree of psychological distress. Research shows that almost half of medical students experience serious psychological distress during their school years [3–5]. Psychological distress is a risk factor that leads to sleep problems [6, 7], internet addiction [8], suicide risk [9, 10], substance abuse [11] and academic performance decline in medical students [12]. Intense psychological distress also interferes with medical students’ studies and lives, reduces their quality of life [13], and even affects their future medical service quality [14]. Therefore, it is essential to explore the predictive factors of medical students' psychological distress (anxiety, depression and stress), which will facilitate intervention and the treatment of medical students' psychological distress and improve their health.

The level of self-efficacy is the core factor that determines an individual's thinking mode, behaviour mode and emotive response in stressful situations [15]. It is found that self-efficacy plays a very important role in the education of medical students [16]. Self-efficacy is related to medical students' motivation, professional commitment, academic achievement and depression [17–19]. Therefore, it is very important to explore how self-efficacy affects the life of medical students. Regulatory emotional self-efficacy embodies the role of self-efficacy in the process of emotional self-regulation, which refers to the subjective evaluation of an individual's ability to express positive emotions in response to positive events and manage undesirable emotions in times of adversity [20]. Individuals who believe that they have the ability to adjust their emotions will make active attempts to do so several times and finally gain more emotional experiences that are more beneficial to them by constantly trying to master emotional adjustment strategies [21]. An experimental study found that compared to a control group, participants who believed their regulatory emotional self-efficacy to be enhanced had a relatively less negative emotional response to negative emotions [22]. Other cross-sectional studies have shown that regulatory emotional self-efficacy can negatively predict depression [23, 24], posttraumatic stress disorder [25], academic stress [26] and trait anxiety [27]. Therefore, it can be inferred that medical students with high regulatory emotional self-efficacy experience less psychological distress, but the influencing mechanism between regulatory emotional self-efficacy and psychological distress has not yet been determined.

Emotions are closely related to an individual's social system and have important communication value [28]. Different emotions convey unique information through the process of communication. Many psychological troubles are caused by interpersonal problems. Anxiety, depression and stress are always accompanied by interpersonal problems [29–31]. According to the theory of the interpersonal relationships of depression, depressed individuals exhibit more limitations in interpersonal problem solving ability and more interpersonal maladjustment [32, 33]. Therefore, difficulties and obstacles experienced in interpersonal relationships are important reasons for psychological distress. Studies have found that interpersonal psychotherapy, as a widely used psychotherapy method, has a positive effect in relieving psychological distress [34]. The interpersonal ability of medical students is related to whether they can become qualified doctors in the future. It is difficult for medical students who do not have interpersonal communication skills to establish a good doctor-patient relationship [35]. On the other hand, the main influencing factor of self-efficacy is the experience of success or failure, and considerable experience with regulating emotions is acquired through interpersonal interaction, so individuals with a high level of self-efficacy experience more harmonious interpersonal relationships [36, 37]. It has also been found that regulatory emotional self-efficacy can predict interpersonal adaptation, and people who are confident in their emotional regulation capacities have stronger communication skills [38]. Therefore, interpersonal adaptation may play an intermediary role between regulatory emotional self-efficacy and psychological distress.

Among the internal factors that affect psychological distress, the degree of self-acceptance is an important factor. Self-acceptance occurs when an individual makes an objective and reasonable evaluation of his own advantages and disadvantages, confirms his own value, and fully accepts his own reality [39]. It is found that medical students with high self-acceptance scores are also more inclined to accept others, which will help doctors and patients form a trusting relationship [40]. Studies have proven that self-acceptance is a predictor of psychological distress [41] in that it can protect individuals from symptoms of depression [42, 43] and significantly predict stress and anxiety levels [44, 45]. Improving self-acceptance of individuals through group activities can help them reduce symptoms of depression and stress [46]. On the other hand, persons with a high level of self-efficacy exhibit greater self-acceptance [47, 48], and self-efficacy can positively predict the level of self-acceptance [49]. Other studies have found a strong correlation between self-acceptance and expectations of emotional regulation [50]. Therefore, it is inferred that self-acceptance may play an intermediary role between regulatory emotional self-efficacy and psychological distress.

**Fig. 1:**
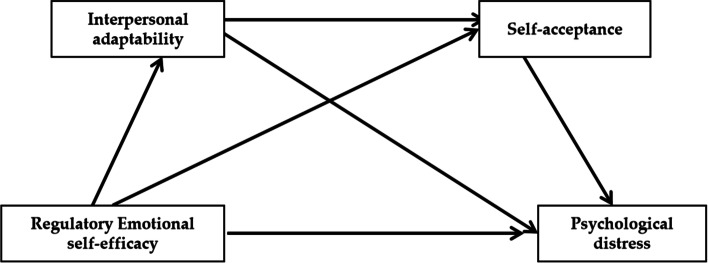
The conceptual model

According to the theory of interpersonal relationships of depression, people who experience more interpersonal rejection and conflict are more likely to form negative self-evaluations, which ultimately increases the risk of experiencing depression [32]. It has also been found that the more negative interpersonal relationships are, the lower levels of self-acceptance are, which is accompanied by greater anxiety and depression [44]. It can be inferred that the level of interpersonal adaptation can affect psychological distress through self-acceptance. Regulatory emotional self-efficacy is also closely related to interpersonal adaptation [38]. Therefore, it can be speculated that emotional self-efficacy negatively predicts psychological distress through the chain mediation of interpersonal adaptation and self-acceptance. The present study was designed to test the following research hypotheses and conceptual model (see Fig. 1):

Based on the foregoing, the following research hypotheses are proposed: H1: interpersonal adaptation among medical students is the mediating factor of regulatory emotional self-efficacy affecting psychological distress; H2: self-acceptance in medical students is the intermediary factor of regulatory emotional self-efficacy affecting psychological distress; H3: regulatory emotional self-efficacy in medical students affects psychological distress through the chain mediation of interpersonal adaptation and self-acceptance. In this study, intermediary analysis was used to explore the relationships between medical students' regulatory emotional self-efficacy, interpersonal adaptation, self-acceptance and psychological distress.

## Methods

### Participants

We conducted the study at Baotou Medical College in Inner Mongolia, China from May 2021 to July 2021. Students' provided data through the Tencent questionnaire platform between their public courses. A total of 570 questionnaires were distributed, and 539 questionnaires (94.6%) were effectively collected. As our exclusion criterion, the questionnaire had to be completed in less than 2 min. The participants included 169 men (31.4%) and 370 women (68.6%) aged between 17 and 22 years old (M = 19.26, SD = 0.93). 192 (35.6%) were first year students,166 (30.8%) were second year students, 116 (21.5%)were third year students, 65(12.1%) were fourth year students. In total, 164(30.4%) of the participants were from cities,169(31.4%) were from towns and 206(38.2%) were from rural areas and 229 (42.5%) were only children while 310 (57.5%) were not only children.

All participants were selected using the cluster random sampling method, who were asked to sign informed consent forms. They were informed that they could withdraw from the study at any time, and were assured that data were anonymous. The study had received ethical approval from the Ethics Committee of Baotou Medical College (3/4 2021).

### Measures

#### Interpersonal adaptability

The Interpersonal Adaptability Scale was compiled by Lu Xiefeng (2003) [51]. It includes 11 questions administered through a single-dimensional questionnaire. An example item is “I find people around me difficult to get along with”. Among the items, 1, 3, 4, 5, 8 and 10 are reverse scores. All the items were rated on a 5-point Likert scale, ranging from 1 = very nonconforming to 5 = very conforming. The higher a score is, the more interpersonal adaptability a college students exhibits. This measure has demonstrated good reliability among college students [52]. In the present study, the Cronbach’s alpha of this questionnaire was measured as 0.88.

#### Regulatory emotional self-efficacy

The Regulatory Emotional Self-efficacy Questionnaire was compiled by Caprara et al. and revised by Yu Guoliang et al. (2009) [20, 53]. The questionnaire includes 12 questions about expressing positive emotions (four items, e.g.,”When something pleasant happens, I will express my pleasure”), regulating depression (five items, e.g.,”I can keep myself away from depression when I am lonely”)and regulating anger (three items, e.g.,”I can avoid getting annoyed when others are deliberately picking on me”). Responses are made on a 5-point Likert scale, ranging from 1 = very nonconforming to 5 = very conforming. The higher a score is, the better regulatory emotional self-efficacy is. This measure has demonstrated good reliability among college students [54]. In the present study, the values of Cronbach’s alpha of this scale and three subscales (i.e., expressing positive emotions, regulating depression and regulating anger) were 0.89,0.85,0.85, and 0.81.

#### Self-acceptance

The Self-acceptance Questionnaire was designed by Zhong Wen and Gao Wenfeng (1999) [55] and includes 16 questions on the two dimensions of self-acceptance(eight items, e.g.,”My inner wish never dares to say it”) and self-evaluation(eight items, e.g.,”I am almost all advantages”). Participants were asked to evaluate themselves, and answers were scored on a 4-point Likert scale, ranging from 1 = strongly disagree to 4 = strongly agree. The higher the total score is, the higher the level of self-acceptance is. This measure has demonstrated good reliability among college students [56]. In the present study, the Cronbach’s alpha of this questionnaire and two subscales (i.e., self-acceptance and self-evaluation) was measured as 0.86,0.85, and 0.84.

#### Psychological distress

Psychological distress was measured by the simplified Chinese version of the Depression-Anxiety-Stress Scale (DASS-21) compiled by Lovibond et al. and introduced by Gong Xu et al. (2010) [57]. The questionnaire includes 21 questions about expressing depression (seven items, e.g.,”I don’t seem to feel happy or comfortable at all”), anxiety(seven items, e.g.,”I feel thirsty”) and stress(seven items, e.g.,”I find it hard to calm myself down”), measured across 4 grades (from 0 = nonconforming to 3 = always conforming). The higher a score is, the more serious psychological distress is. This measure has demonstrated good reliability among college students [58]. In the present study, the Cronbach’s alpha of this questionnaire and three subscales (i.e., depression, anxiety and stress) was measured as 0.89, 0.80, 0.71, and 0.78, respectively.

### Data analysis

SPSS22 statistical software was used to sort and analyse the data. The statistical methods used include descriptive analysis, Pearson correlation analysis, and mediation effect tests completed with Process plug-in (Model 6) [59].Mediation hypotheses were tested with bootstrapping via a resampling of 5000 samples to calculate 95% confidence intervals (CIs). If the 95% CI did not contain zero and the *p* value was < 0.05, results were deemed statistically significant.

## Results

### Descriptive and correlation analysis

Descriptive and correlation analysis were performed on interpersonal adaptability, regulatory emotional self-efficacy, self-acceptance and psychological distress. The results are shown in Table 1. Regulatory emotional self-efficacy, interpersonal adaptability and self-acceptance are negatively correlated with psychological distress (*r* = -0.39, -0.59, and -0.53) to a moderate degree [60]. Self-acceptance is moderately positively correlated with interpersonal adaptability (*r* = 0.65) and regulatory emotional self-efficacy (*r* = 0.49), and interpersonal adaptability is also moderately positively correlated with self-acceptance (*r* = 0.53).

**Table 1:** Descriptive statistical results and correlation analysis (*n* = 539)

Variable	Test Score	Correlation Coefficient(r)			
	**(mean ± SD)**	**1**	**2**	**3**	**4**
1. regulatory emotional self-efficacy	43.05 ± 6.21	-			
2. Interpersonal adaptability	42.50 ± 7.00	0.53**	-		
3. Self-acceptance	41.73 ± 6.61	0.49**	0.65**	-	
4. Psychological distress	6.56 ± 6.84	-0.39**	-0.59**	-0.53**	-

^**^p < 0.01

### Multiple mediation analysis

Using the Harman single-factor test method, an exploratory factor analysis of all measurement items without rotation was carried out. The results show 13 common factors with characteristic values of greater than 1, and the first common factor explains the critical standard of 25.61% of the total variance and less than 40% [61]. This shows an absence of common method bias in this study.

Model 6 of the SPSS macro compiled by Hayes was used to test the mediating effect of interpersonal adaptation and self-acceptance on the relationship between regulatory emotional self-efficacy and psychological distress. The results (see Table 2) show that medical students' regulatory emotional self-efficacy significantly positively predicts interpersonal adaptation (β = 0.53, *P* < 0.001) and self-acceptance (β = 0.20, *P* < 0.001). Interpersonal adaptation significantly positively predicted self-acceptance (β = 0.55, *P* < 0.001). When all predictive variables are included in the regression equation at the same time, only interpersonal adaptation (β = -0.41, *P* < 0.001) and self-acceptance (β = -0.23, *P* < 0.001) have a significant negative predictive effect on psychological distress, while regulatory emotional self-efficacy (β = -0.06, *P* = 0.16) has no predictive effect on psychological distress.

**Table 2:** Results of the multiple mediation analysis

Regression Model Outcome Variable	Predictor Variable	Goodness-of-Fit Indices			Regression Coefficient and Significance	
		***R***	***R***^***2***^	***F***	***β***	***t***
Interpersonal adaptability		0.54	0.29	110.77***		
	Gender				-0.18	-2.32*
	Emotional self-efficacy				0.53	14.64***
Self-acceptance		0.68	0.46	150.42***		
	Gender				0.04	0.52
	Interpersonal adaptability				0.55	5.39***
	Emotional self-efficacy				0.20	14.50***
Psychological distress		0.63	0.39	85.95***		
	Gender				-0.19	-2.54*
	Interpersonal adaptability				-0.41	-8.70***
	Self-acceptance				-0.23	-5.11***
	Emotional self-efficacy				-0.06	-1.42

^*^p < 0.05, ***p < 0.001

**Fig. 2:**
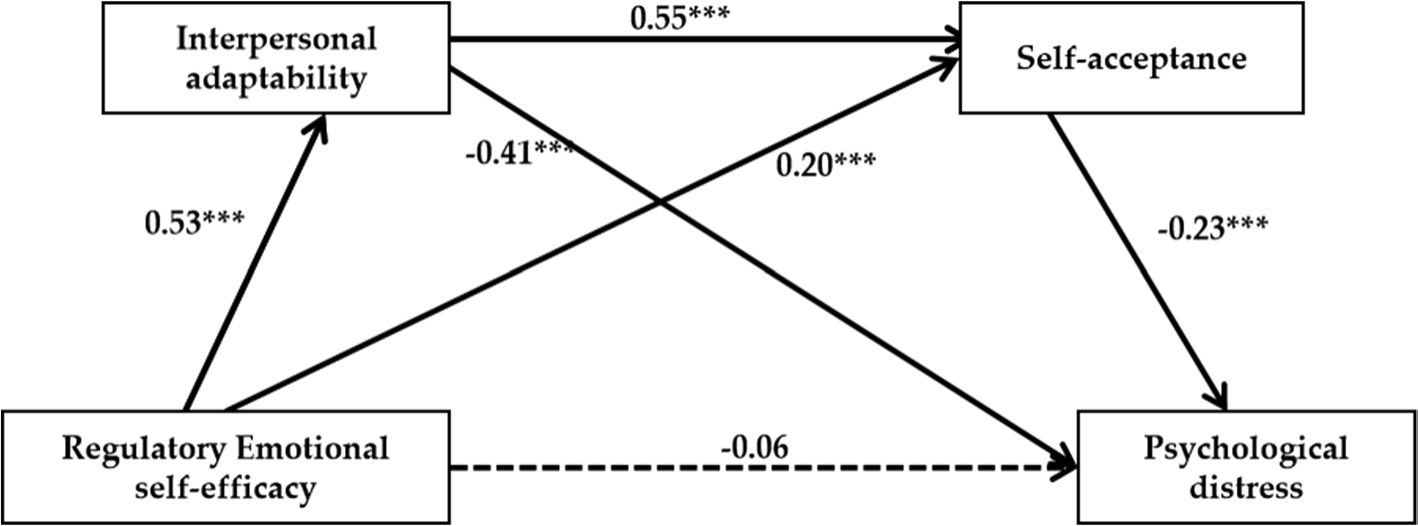
Results of the multiple mediation analysis. *** *p* < 0.001. *N* = 539

The mediating effect test results show that (see Table 3), the bootstrap 95% confidence interval of the indirect effects of interpersonal adaptation and self-acceptance does not contain 0, which indicates that interpersonal adaptation and self-acceptance are mediating variables of regulatory emotional self-efficacy affecting psychological distress with a total mediating effect value of -0.33, accounting for 84.62% of the total effect (-0.39). Specifically, three paths are found: first, the indirect effect of regulatory emotional self-efficacy → interpersonal adaptation → psychological distress (effect value-0.22); second, the indirect effect of regulatory emotional self-efficacy → interpersonal adaptation → self-acceptance → psychological distress (effect value-0.06); and third, the indirect effect of regulatory emotional self-efficacy → self-acceptance → psychological distress (effect value -0.05). The model is presented in Fig. 2.

**Table 3:** Bootstrap analysis of multiple mediation effects

Effect	Effect Size	SE	95% CI Boot CI		Percentage of Effects
			**Lower**	**Upper**	
Total effects	-0.43	0.04	-0.52	-0.35	100%
Direct effects	-0.06	0.05	0.16	-0.15	13.95%
Total mediation effects	-0.37	0.05	-0.47	-0.28	86.05%
Regulatory emotional self-efficacy → Interpersonal adaptability → Psychological distress	-0.24	0.04	-0.33	-0.17	55.81%
Regulatory emotional self-efficacy → Interpersonal adaptability → Self-acceptance → Psychological distress	-0.08	0.02	-0.12	-0.04	18.60%
Regulatory emotional self-efficacy → Self-acceptance → Psychological distress	-0.05	0.02	-0.09	-0.02	11.63%

*N* = 539.Bootstrap = 5000. →  = unidirectional path

## Discussion

The results of this study confirm the following hypothesis: Regulatory emotional self-efficacy affects psychological distress in medical students through interpersonal adaptation and self-acceptance. This role includes three indirect paths: the single intermediary role of interpersonal adaptation, the single intermediary role of self-acceptance, and the chain intermediary role of interpersonal adaptation and self-acceptance, among which the path of interpersonal adaptation has the largest effect value. To the best of our knowledge, this mediation model has not been verified in previous studies. These findings help us further understand the mechanism by which medical students' regulatory emotional self-efficacy affects psychological distress and can help us better treat and prevent medical students' psychological distress.

First, interpersonal adaptation plays an intermediary role between medical students' regulatory emotional self-efficacy and psychological distress, which proves the authenticity of H1, which means that medical students with poor interpersonal adaptation are more likely to experience psychological distress when their level of emotional self-efficacy is low. Low-level regulatory emotional self-efficacy leads to an increase in psychological distress, which is consistent with previous results [23–27]. Individuals with high levels of regulatory emotional self-efficacy are more confident in coping with their emotions, especially negative emotions, and they adopt effective emotional regulation strategies to change the adverse effects of negative events [62]. Therefore, in the face of interpersonal difficulties, individuals with high levels of regulatory emotional self-efficacy can adjust their emotions in time and adapt faster, which has been proven by previous studies [38]. On the other hand, good personal relationships can improve negative emotions and enhance self-efficacy [63]. It is also found that the interpersonal relationship of medical students is related to mental health  [64, 65], and the bad interpersonal relationship is accompanied by more depression [66]. Therefore, promoting interpersonal adaptation may help directly or indirectly reduce psychological distress in medical students. Research shows that medical students are under great academic pressure, and suffer more psychological problems than the general population and college students in other fields [1]. Managers of medical colleges should pay attention to the interpersonal adaptation of medical students, because the ability of interpersonal communication and communication has become the core competence of medical graduates all over the world [67], which is related to the quality of doctor-patient relationship in future work. It is difficult for students who have difficulty in interpersonal adjustment to master this ability. This study provides a solution to help medical students improve their ability to regulate emotions and make them believe that they have the ability to deal with various emotions. An intervention study found that teaching intervention can significantly improve college students' regulatory emotional self-efficacy [68]. A strong sense of emotional self-efficacy can help students improve their interpersonal adaptability, acquire richer interpersonal skills and help them prevent or manage psychological problems. In addition, some studies have found that group interpersonal therapy is often used to improve the interpersonal skills of college students, and the effect is remarkable, which also provides us with a method to improve the interpersonal adaptation of medical students [69].

Second, self-acceptance plays an intermediary role between medical students' emotional self-efficacy and psychological distress, which proves the authenticity of H2. that is, lower emotional self-efficacy is accompanied by a lower degree of self-acceptance among medical students, which leads to more serious psychological distress. Some studies have shown that individuals who lack confidence in their own skills tend to exaggerate their shortcomings, deny their value and refuse to accept themselves [47, 48]. Individuals with little self-acceptance easily to retreat when encountering problems and are more likely to fail when facing challenges, which will spur more psychological difficulty. Because the higher the level of self-acceptance, the lower the individual's anxiety, depression and stress [44, 70, 71], and some researchers use self-acceptance group counseling to help teenagers relieve depression [46]. Therefore, it is of great significance to promote the self-acceptance level of medical students to reduce their psychological distress. In addition, medical students with high self-acceptance level are more receptive to others, which will help them form a good relationship with patients [40]. It has been found that painting therapy, dance therapy, group therapy, cognitive behaviour therapy and hypnotherapy are effective in improving self-acceptance [46, 72–74]. Health workers can consider combining these methods to improve self-acceptance in medical students and reduce the influence of psychological distress on these students.

Finally, the results of this study show that when interpersonal adaptation and self-acceptance are included the regression equation, the direct predictive effect of emotional self-efficacy on psychological distress becomes insignificant. Moreover, the results of our intermediary test show that the chain intermediary function of interpersonal adaptation and self-acceptance also serves as an important mean through which emotional regulation self-efficacy affect medical students' psychological distress. A higher level of emotional self-efficacy can help individuals better achieve interpersonal adaptation [38]. Individuals with good interpersonal relationships usually have higher levels of self-acceptance and experience less anxiety and depression [44]. Therefore, students with high self-efficacy in emotional regulation are less likely to face challenges when facing interpersonal difficulties and more likely to mobilize emotional regulation resources, respond positively, understand themselves and accept themselves in practice. Self-accepting students are more likely to experience psychological harmony, have more satisfaction with life and experience fewer psychological difficulties [75]. Thus, in order to reduce medical students' psychological problems, improve their mental health status and be better qualified for medical work in the future, medical educators should start from three aspects: emotion regulation, self-efficacy, interpersonal adaptation and self-acceptance, so as to comprehensively improve medical students' abilities in these aspects.

Research also has some limitations. Although the model verified in this study is based on the existing research and theoretical basis, it is limited by questionnaire and cross-sectional study, so it is impossible to infer the exact causal relationship. In the future, longitudinal study can be used to investigate the relationship between them. In addition, the self-assessment method is used to investigate the subjects in this study, and the results may be influenced by the reaction deviation of the subjects. Finally, this study selects medical students from the same medical college as the research object, which means that the representativeness of the sample is biased.

## Conclusions

This study provides researchers with the overall relationship between emotional self-efficacy and psychological distress. Those medical students who believe that they can adjust their emotions and have good interpersonal adaptability and self-acceptance will face less depression, anxiety and pressure, and at the same time, they will be easier to communicate with patients efficiently and achieve a good doctor-patient relationship. Future research can combine teaching intervention, cognitive behaviour therapy and group interpersonal therapy to formulate a holistic intervention plan for medical students' emotional self-efficacy, self-acceptance and interpersonal adaptation, and verify the effectiveness of the plan in reality.

## Data Availability

The datasets generated and analysed during the current study are not publicly available because they contain medical student information that they did not consent to have shared publicly at the individual level but aspects of the dataset may be available from the corresponding author on reasonable request.

## Abbreviations

DASS 21: Depression Anxiety Stress Scale
